# Probing the behavior and kinetic studies of amphiphilic acrylate copolymers with bovine serum albumin

**DOI:** 10.1038/s41598-023-27515-5

**Published:** 2023-03-20

**Authors:** Shehla Mushtaq, Muhammad Asad Abbas, Habib Nasir, Azhar Mahmood, Mudassir Iqbal, Hussnain A. Janjua, Nasir M. Ahmad

**Affiliations:** 1 Department of Chemistry, School of Natural Sciences (SNS), National University of Sciences and Technology, H-12, Islamabad, 44000 Pakistan; 2 Chemical Engineering & Material Science, Michigan State University, East Lansing, MI 48824 USA; 3 Polymers Research Lab, Polymers and Composites Research Group, School of Chemical and Materials Engineering (SCME), National University of Sciences and Technology, H-12, Islamabad, 44000 Pakistan; 4 Department of Industrial Biotechnology, Atta-Ur-Rahman School of Applied Biosciences (ASAB), National University of Sciences and Technology, H-12, Islamabad, 44000 Pakistan

**Keywords:** Chemistry, Polymer chemistry, Polymer synthesis

## Abstract

This article presents that acrylate copolymers are the potential candidate against the adsorption of bovine serum albumin (BSA). A series of copolymers poly(methyl methacrylate) (*p*MMA), poly(3-sulfopropyl methacrylate-co-methyl methacrylate) *p*(SPMA-co-MMA), and poly(dimethylaminoethyl methacrylate-co-methyl methacrylate) *p*(DMAEMA-co-MMA) were synthesized via free radical polymerization. These amphiphilic copolymers are thermally stable with a glass transition temperature (Tg) 50–120 °C and observed the impact of surface charge on amphiphilic copolymers to control interactions with the bovine serum albumin (BSA). These copolymers *p*MD1 and *p*MS1 have surface charges, − 56.6 and − 72.6 mV at pH 7.4 in PBS buffer solution that controls the adsorption capacity of bovine serum albumin (BSA) on polymers surface. Atomic force microscopy (AFM) analysis showed minimum roughness of 0.324 nm and 0.474 nm for *p*MS1 and *p*MD1. Kinetic studies for BSA adsorption on these amphiphilic copolymers showed the best fitting of the pseudo-first-order model that showed physisorption and attained at 25 °C and pH 7.4 within 24 h.

## Introduction

Protein interaction and adsorption on the surface of polymeric materials affect the functionality of materials and their performance. Protein adsorption on polymeric materials has biological applications and demand while the dispersal of these proteins played a substantial role in the biological response^1–4^. Morphology and characteristics of the material surface control the deposition and film formation with intervening protein^5–8^. Poly methyl methacrylate with its unusual physicochemical qualities and structural characteristics has attracted a lot of interest as a building block for the production of hybrid polymeric materials^9^. PMMA is utilized in a variety of medical devices, including blood pumps and dialyzers, due to its superior biocompatibility, hemocompatibility, and simplicity of manipulation^10^. The glass transition temperature (*T*_g_) of MMA after copolymerization with hydrophilic monomers, as well as hydrogen-bonding interactions between these two monomer segments, improved its thermal stability^11,12^. These thermally stable amphiphilic copolymers are useful in controlling protein adsorption and can be helpful in degrading the analytical performance of devices and body implants^13^. Designing material for protein adsorption, on the other hand, is significantly more difficult, thus the adsorbent material should be thoroughly defined^14^. The conversion of hydrophobic polymers to amphiphilic and hydrophilic is a common, albeit non-restrictive theme among the strategies of resistance towards protein adsorption^15,16^. Biological molecules interaction and film formation on materials controlled via charge-like proteins undergo a conformational change that associate with material and its amphiphilic, hydrophobic and hydrophilic domain^17–19^.

BSA protein is highly demanded by researchers and frequently used because of its high purity and water solubility^20^. BSA has been the most studied protein in adsorption–desorption process as a function of charge, protein concentration, pH, substrate charge and polarization potential, permitting studies in the presence and absence of charge^21,22^. Bovine serum albumin (BSA) has poorer internal stability than other proteins, it was selected as the model protein for the adsorption studies^23^. Higher internal stability proteins exclusively adsorb onto hydrophilic surfaces via electrostatic interactions, whereas lower internal stability proteins adsorb onto any surface independent of electrostatic connections^24^. However, because of the complex structure of protein and the sort of interaction it involves, the surface quality of the adsorbent material is critical^25^.

Inspired by these properties to control protein adsorption on the surface of polymers we synthesized the amphiphilic copolymers changing the concentration of different monomers. These acrylate copolymers showed good antifouling properties through controlling their surface energies as reported in our previous work^26–30^. Amphiphilic acrylate copolymers are obtained through direct polymerization of hydrophobic monomers with hydrophilic monomers by radical polymerization^31,32^. Zho et al. synthesized a number of amphiphilic copolymers by radical polymerization of 2-hydroxyethyl methacrylate and 2-perfluorooctylethyl. These polymers have better antifouling properties as compared to homopolymers when they have 4–7 and 4–14% hydrophobic and hydrophilic groups on their surface^33^. Yufei Wang et al. and Befeng et al. have studied antifouling and adsorption properties but through the modification of polymeric surfaces by embedding different groups^16,22^.

In this study, intrinsic amphiphilic copolymers composed of hydrophobic and hydrophilic acrylate monomers that were synthesized by single-step free radical polymerization. A series of amphiphilic compositions was prepared by varying the monomers ratio of SPMA, DMAEMA and MMA in copolymers. The synthesis of homopolymer and copolymers was confirmed by FTIR and ^1^H-NMR while thermal stability of the polymers was examined by TGA and DSC. In this study bovine serum albumin protein was selected as a model protein to check the adsorption on the surface of polymers. Protein film formation on the surface of these copolymers was characterized by SEM and AFM. Zeta potential of copolymers was performed to measure surface charge of amphiphilic copolymers at polymers constant temperature 25 °C and pH 7.4. Protein adsorption on the surface of polymers was physiosorption that confirmed after obeying pseudo first order kinetics, adsorption capacity (Q_o_) was found maximum, 9 mg/g for *p*MMA and minimum 0.9 mg/g for *p*MD1 through UV–Vis spectroscopy.

## Materials and method

### Materials

All chemicals were of analytical grade and used in chemical synthesis without further purification. Dimethyl aminoethyl methacrylate (DMAEMA, 98%) (Sigma-Aldrich, Germany), methyl methacrylate (MMA, 99%) (Sigma, USA), 3-sulfopropyl methacrylate (SPMA 99%) (Sigma-Aldrich, Germany) 2,2-azobisisobutyronitrile (AIBN, 98%) (Sigma, USA), N,N-dimethyl formamide (DMF, 99%) (Aldrich, USA), ethanol, phosphate buffer solution (PBS) (Amersco, Belgium) and bovine serum albumin (BSA) (Aldrich, USA).

### Method

Both the homopolymer and copolymers were synthesized through free radical polymerization with 62–65% yield^26,30^.

### Synthesis of homopolymer

*p*MMA was synthesized under inert atmosphere via free radical polymerization in DMF solvent in the presence of 2,2′-azobisisobutyronitrile (AIBN) as initiator at 70 °C. Molar ratios of monomer, solvent and initiator is 10:100: 0.02. MMA (10 g, 99.9 mmol), were introduced in the reaction flask. The polymerization process was allowed to run for 5 h with continuous stirring under nitrogen purging through the Schlenk line at 70 °C.

### Synthesis of copolymers

**Figure 1:**
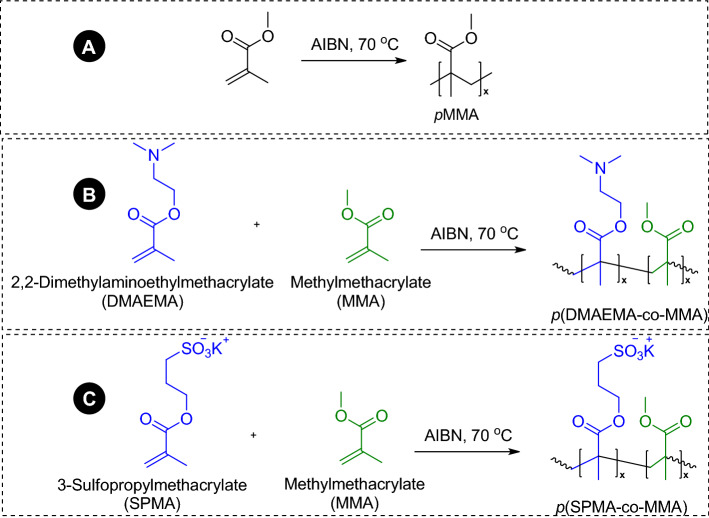
Synthesis of (**A**) pMMA; (**B**) p(DMAEMA-co-MMA) and (**C**) p(SPMA-co-MMA).

Synthesis of amphiphilic copolymers of *p*(MMA-co-SPMA) and *p*(MMA-co-DMAEMA) was done by free radical polymerization. Copolymerization was done by using the molar ratio of 10:100:0.02 of monomer:solvent:initiator in inert atmosphere at 70 °C in the reaction flask (Fig. 1). Polymerization was done with continuous nitrogen gas purging and stirring for 5 h.

### Instrumentations used for characterization

A Bruker ALPHA-P FTIR equipment was utilized to identify the functional groups. Bruker, ASCEND 400-MHz NMR spectrometer was utilized to collect ^1^H-NMR spectra^30^. TGA was done by using a Mettler Toledo STARe thermogravimetric system in a N_2_ atmosphere throughout a temperature range of 0–550 °C at a scan rate of 10 °C/min and 10–12 mg polymer samples were used. DSC was performed by a Mettler Toledo DSC STAR system under nitrogen purge and hermetic pans were used with sample weight of 10–12 mg. A Jasco UV–Vis (model V-530) spectrophotometer was used to measure the concentration of BSA in solution. For UV phosphate buffer solution was used to maintain pH 7.4, polymer and BSA ratio were taken 6 g/L and 0.60 g/L. Systronic microprocessor pH meter (model l-362) was used for the measurement of pH. Scanning electron microscope (JSM-7500F, JEOL Ltd., Japan) operated in secondary electron mode at a 5 kV acceleration voltage was used to examine the surface morphology of polymers. Before scanning electron microscopy (SEM) samples were dried and there was no moisture. Sample for SEM prepared through platinum sputtering and then analyzed for morphology after adsorption. Atomic force microscopy, AFM (Asylum—Cypher™ AFM) was used to measure the roughness caused by BSA adsorption. All samples with size 1 × 1 used and samples were dried before performing AFM. Zeta plus analyzer (Malvern) was used to measure the zeta potential of polymer solutions with concentration of 0.02 wt% in water by electrophoresis light scattering at pH 7.4 and 25 °C.

### Adsorption of BSA

The BSA protein adsorption studies were performed in a batch process of adsorption for specific contact time at 25 °C at pH 7.4 in phosphate buffer solution. Here polymer and BSA ratio were taken 6 g/L and 0.60 g/L, after centrifugation the solvent was filtered and then analyzed using the Lowery technique. The adsorption efficiency of the prepared materials was calculated using the following equations:

1 $$\left(qe\right)= \frac{[\left(Co-Ce\right) x V}{(m)}$$

2 $$\frac{Ce}{Qe} = \frac{1}{{K}_{L}x {q}_{m}} + \frac{Ce}{{q}_{m}}$$

3 $$\mathrm{log}\left(q\right)=\mathrm{log}\left({K}_{f}\right) + \frac{1}{n}(Ce)$$

where “qe” is the adsorption capacity of BSA (mg/g), “V” is the volume of solution (L), “m” is the mass of adsorbent (g), and “Co” and “Ce” are the initial and final BSA concentrations (mg/L), respectively. The process of adsorption has also been expressed by Langmuir and Freundlich relations [Eqs. (1) and (2)]. The logarithmic form of the Freundlich equation is expressed in Eq. (3), where “Kf” having unit (mg/g) is the Freundlich constant and “n” is the Freundlich exponent.

4 $$\mathrm{ln}\left({q}_{e} - {q}_{t}\right) = \mathrm{ln}\left({q}_{e}\right) - {K}_{1}t$$

5 $$\frac{t}{{q}_{t}} = \frac{1}{{K}_{2}{q}_{e}^{2}} + \frac{1}{{q}_{e}}t$$

To explore BSA adsorption kinetics, pseudo first order and pseudo second order kinetics [Eqs. (4) and (5)] were also applied, whereas Eq. (6) was used to investigate the maximum adsorption.

The BSA concentration of the adsorbent phase (qe, mg/g) was determined using,

6 $$qe=\frac{(Co-C)}{w}$$

where “qe” is equilibrium adsorption of BSA, “Ci” and “Ce” are initial and equilibrium concentration (mg/L), respectively, “V” is the volume of solution (L), and “W” is the weight of dry adsorbent (g)^34^.

### Ethical approval

This study does not involve any experimentation related to vertebrates and invertebrates.

## Results and discussion

### Infrared spectroscopy

**Figure 2:**
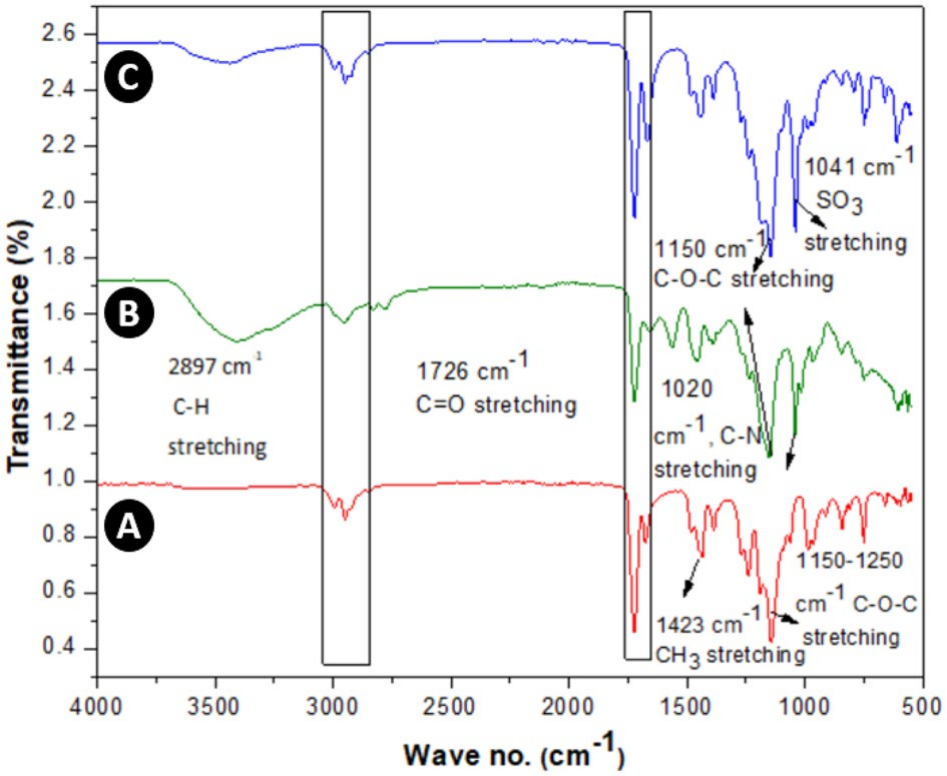
FTIR spectra of polymers *p*MMA, *p*MD1 and *p*MS1.

FTIR analysis of the polymers *p*MMA, *p*MD1 and *p*MS1 is shown in Fig. 2. Here the band at 1433 cm^−1^ (spectrum A) was assigned to the asymmetric bending vibration of the *p*MMA CH_3_ group^16^. The absorbance value of 1381 cm^−1^ was caused by the deformation of OCH_3_ of *p*MMA^30^. Stretching and bending of C–O–C was represented by the typical signals detected at 1265 cm^−1^ and 1145 cm^−1^, respectively^35^. The band at 1193 cm^−1^ was due to stretching vibration of –OCH_3_ and vibrations of –CH_2_ at 977 cm^−1^ and 716 cm^−1^ were assigned for wagging and rocking modes of *p*MMA, respectively^36^.

The copolymer *p*MD1 in spectrum B, contains the DMAEMA and MMA distinctive bands and for C-N, band at 1020 cm^−1^ assigned to stretching vibration of tertiary amine that confirms the presence of DMAEMA segment into copolymers^35^. MMA moiety for C=O bond belongs to ester that shows absorption band at 1730 cm^−1^, –CH_2_ group gives band at 1450 cm^−1^ for bending vibration^30^. The band at 3000–3500 cm^−1^ is owing to the O–H group due to moisture present in DMAEMA because of its hydrophilic nature as shown in Fig. 2. The stretching vibration at 749 cm^−1^ is due to –CH_2_ group and –SO_3_ showed asymmetric vibration of *p*MS1 in the spectrum C. The bands at 1354 cm^−1^ and 1145 cm^−1^ are due to C–O stretching vibration of the ester^30,35^.

### ^1^HNMR study of copolymers

**Figure 3:**
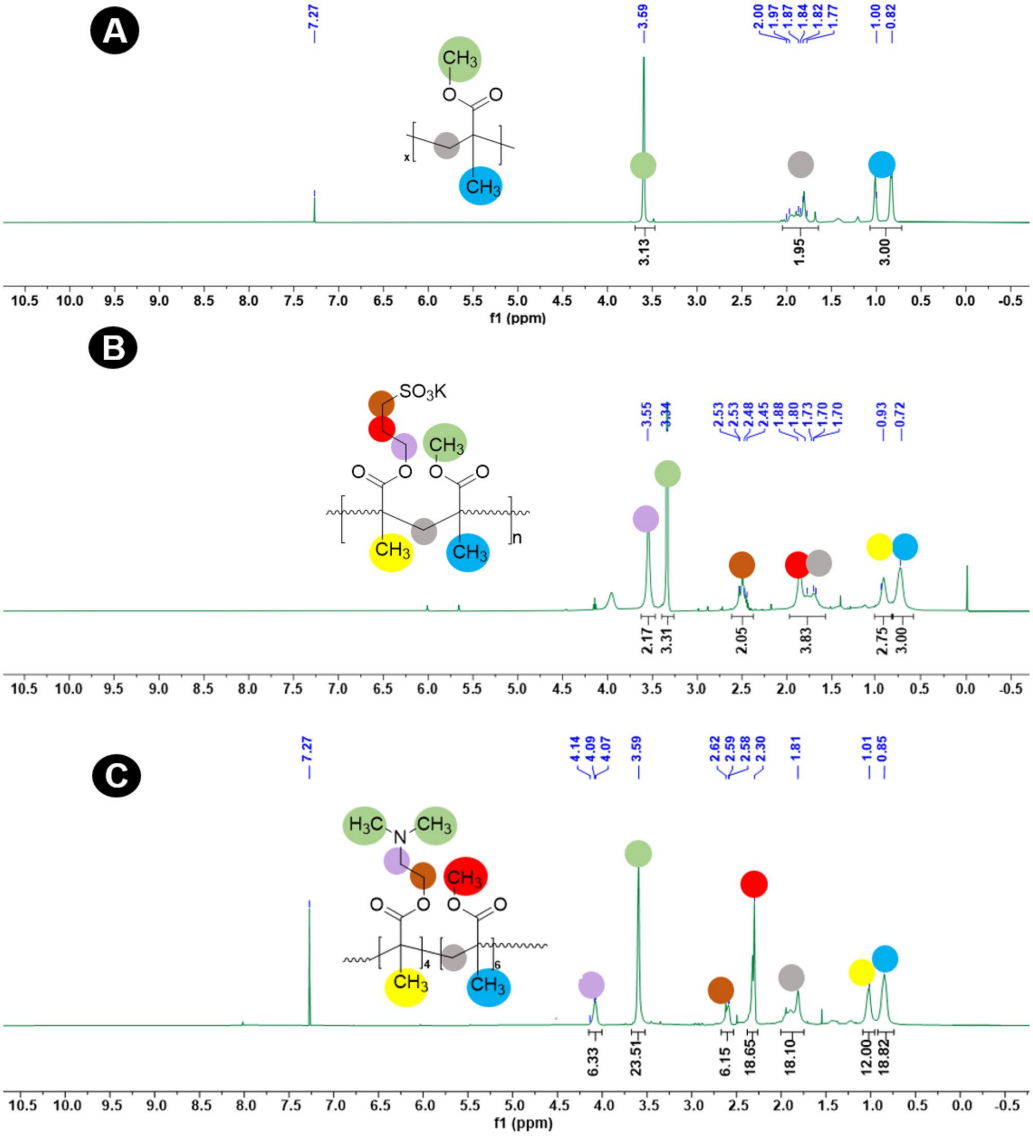
^1^HNMR spectra of polymers: (**A**) *p*MMA; (**B**) *p*MD1 and (**C**) *p*MS1.

The ^1^HNMR spectrum (Fig. 3A) of the homopolymer of *p*MMA shows a signal at 3.6 ppm because of resonance of –OCH_3_ protons^30^.

The ^1^HNMR spectrum of *p*(SPMA-co-MMA) is presented in Fig. 3B that demonstrates the peaks for chemical shifts related to variant protons surroundings in diverse chemical moieties enclosed in the copolymer^37^. The spectrum clearly shows MMA and SPMA signals with significant differences of protons dynamic mobility that validate structure of the copolymer and effect of the association process that displayed spectrum^30^. In the *p*(MMA-co-SPMA) copolymer the peaks at 4.1 ppm and 3.35 ppm correspond to the –OCH_2_ and OCH_3_ protons, respectively. The peak for –CH_2_ protons in the vicinity of -SO_3_K is observed at 2.5 ppm slightly upward for SPMA^38^. Furthermore, CH_2_SO_3_K group shows a signal at 2.72 ppm due to resonance and the peaks at 3.1–3.5 ppm show the indication of copolymer formation^30^. The methyl groups along the chains show peaks at 1.1 ppm and 1.8 ppm that are enclosed by the different environments.

The composition of two monomers in the copolymer, p(MMA-co-DMAEMA) indicate peaks at 4.1 ppm (due to the –OCH_2_ protons in the DMAEMA moiety) and at 3.6 ppm (corresponding to the –OCH_3_ protons in the MMA moiety) (Fig. 3C)^39^. In the copolymers, compositions of the monomers were calculated by division through peak intensities at 2.5 ppm for protons of –OCH_2_ group of DMAEMA to the peak intensity at 2.3 ppm for protons of –OCH_3_ group of MMA. In the diblock the peaks are observed at 4.1 ppm for the protons of N-CH_2_ and for the protons of tertiary amine, –N(CH_3_)_2_ at 3.59 ppm^40^.

### Thermal analysis of polymers

**Figure 4:**
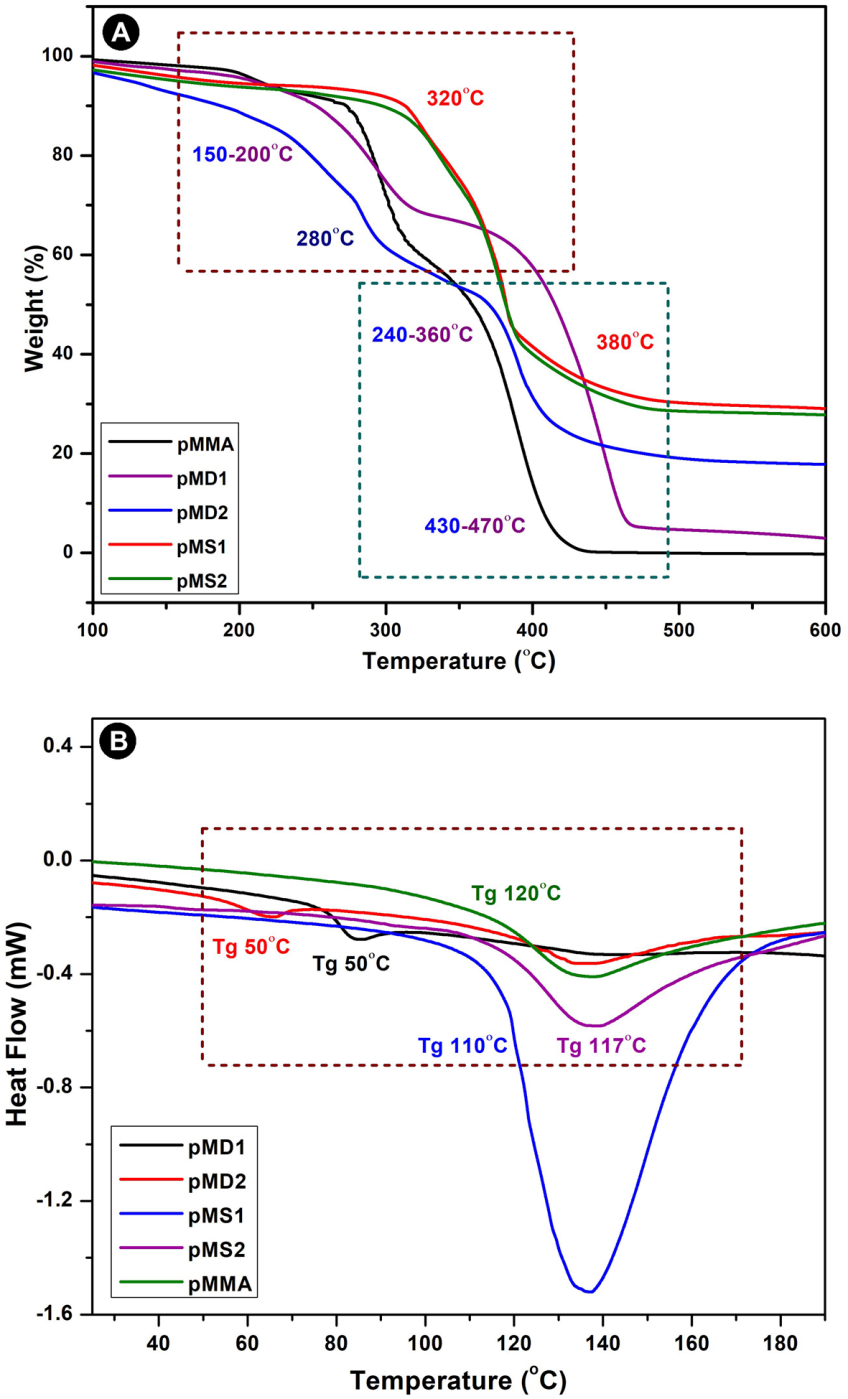
TGA(3A) & DSC (3B) analysis of polymers: *p*MMA, *p*MD1, *p*MD2, *p*MS1 and *p*MS2.

The findings of thermal analysis of *p*MMA, p(MMA-co-DMAEMA) and p(MMA-co-SPMA) by TGA are shown in Fig. 4A. In this thermal study temperature range was up to 550 °C at which *p*MMA was decomposed leaving no residual weight.The pyrolysis of the *p*(MMA-co-SPMA) and *p*(MMA-co-DMAEMA) gave small yields of residual weight. The pyrolysis of the *p*(MMA-co-SPMA) and *p*(MMA-co-DMAEMA) gave small yields of residual weight. These polymers showed weight loss in different stages and different temperatures between 150 and 470 °C^41^. *p*MMA showed first decomposition of 10% at 280 °C, 40% at 380 °C and 100% decomposition at 440 °C. Some research has been conducted to explore the thermal degradation of *p*MMA, which indicates degradation in the first stage between 200 and 280 °C and this was happened because of head to head scission of linkages. The scission in the second stage (300–375 °C) of the unsaturated chain ends due to weight loss and disproportionation occurs 60%^41^.

The copolymer of *p*(MMA-co-DMAEMA) start to melt at temperature of 100 °C and could be attributed to the decomposition with the loss of functionality of end groups above 100 °C. Here 30–40 % weight loss between 220 °C and 270 °C is significantly more than expected due to the end group loss, as stated in previous literature^41^.

At 440 °C, copolymers *p*MD1 and *p*MD2 lost 75 percent of their weight, demonstrating that copolymers are more stable than homopolymers. The decomposition of *p*(MMA-co-SPMA) happens in two stages, the first of which occurs in the temperature ranging from 320 to 360 °C and corresponds to the decomposition of the polymer caused by the breaking of weak head-to-head links, resulting in a 10% weight loss. The second phase occurs in the 360–440 °C range due to the breakdown of the unsaturated polymer chain ends caused by the disproportionation termination reaction. According to Kashiwagi et al., the first step could be attributable to the breakdown of the unsaturated polymer chain ends^42^. The second stage at 380 °C is most likely related to the random breakdown of the polymers major chains.

In *p*(MMA-co-SPMA) copolymers, 60% weight loss was reported, with beginning points of weight loss appearing at higher temperatures than in *p*MMA, and thermal degradation occurs in two phases. Further homopolymer and copolymers were characterized for thermal properties by DSC as shown in Fig. 4B, DSC thermograms of *p*MMA, p(MMA-co-DMAEMA) and p(MMA-co-SPMA) copolymers. The glass transition temperature of pure *p*MMA is 120 °C, which is consistent with the reported *T*_g_ value^11,43^. On copolymerization of MMA with DMAEMA and SPMA obtained single glass transition (*T*_g_) point that indicate copolymers are homogeneous. Lower *T*_g_ recommend the increase in the segmental mobility of the DMAEMA chains in *p*(MMA-co-DMAEMA). *p*MD_1_ and *p*MD_2_ had *T*_g_, 50 °C and 65 °C with varying concentration of DMAEMA and MMA. On the other hand, *p*MS1 and *p*MS2 showed greater *T*_g_ and endothermic flow of heat around 110–117 °C that showed rigidity of copolymers with increasing content of 3-sulfopropyl methacrylate content because of introduction of sulfonyl groups in the copolymers. SPMA concentration into the copolymers enhance the bonding and attraction between the chains of polymer that increase the crystallization and stability of polymers as shown in Table 1.

**Table 1:** TGA and DSC of copolymers.

Samples	Stages of TGA-weight loss temperature (°C)			T_g_ (°C)
	1st stage	2nd stage	3rd stage	
*p*MMA	200–280	280–380	380–480	120
*p*(MMA-co-SPMA)	320–360	360–440	–	110–117
*p*(MMA-co-DMAEMA)	150–200	240–360	370–435	50–65

### Zeta potential of copolymers

**Figure 5:**
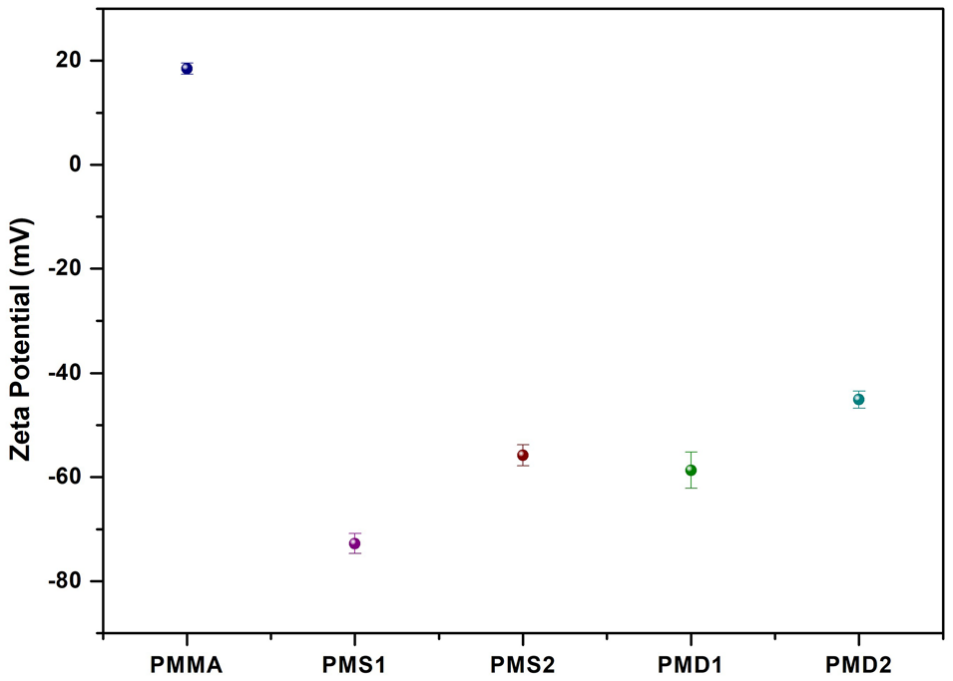
Zeta potential of *p*MMA, *p*MS1, *p*MS2, *p*MD1 and *p*MD2.

Figure 5 shows the zeta potential curves of the *p*MMA, *p*MD1, *p*MD2, *p*MS1 and *p*MS2 in buffer solution at pH 7.4 and 25 °C. Pure *p*MMA shows the zeta potential of 18 mV which implies that pMMA at ionic strength of 0.01 at pH 7.4 is negatively charged^44^. The copolymers of *p*DM1 and *p*DM2 show the zeta potential of − 56.1 and − 45.4 mV that indicates the decrease in zeta potential with the increase in the concentration of DMAEMA (Table 2). DMAEMA is pH responsive and normally it has positive charge due to the amino group. This explains the solubility of DMAEMA in water, as the concentration increases, the polymer chains get more dissociated. *p*SM1 and *p*SM2 showed the zeta potential of -72.2 and -56.6 mV and the increase in zeta potential was due to the increase in the concentration of 3-sulfopropyl methacrylate^45^.

**Table 2:** Concentration of monomers in amphiphilic copolymers, zeta potential (mV), roughness (nm) and BSA adsorption capacity (%).

Samples	Concentration (g)			Zeta potential (mV) at pH 7.4	Roughness, R (nm)
	MMA	DMAEMA	SPMA		
*p*MMA	20	–	–	18 ± 1	6.85
*p*MS1	10	–	10	− 72.7 ± 1	0.324
*p*MS2	14		6	− 56.6 ± 1	1.85
*p*MD1	10	10	–	− 56.1 ± 1	0.474
*p*MD2	14	6	–	− 45 ± 1	0.871

### Morphology of the homopolymer and copolymers

**Figure 6:**
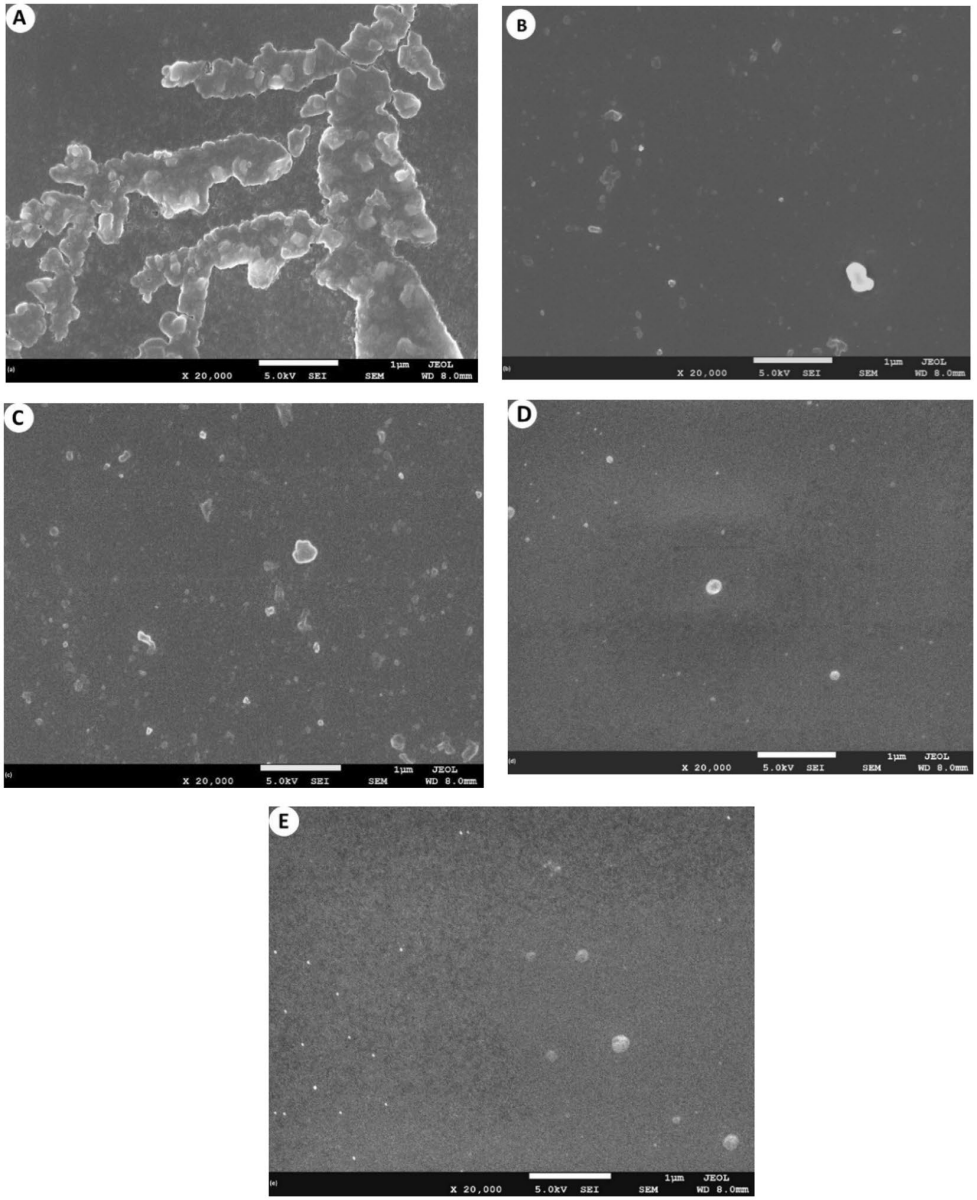
SEM of polymers, (**A**) *p*MMA; (**B**) *p*MS1; (**C**) *p*MS2; (**D**) *p*MD1 and (**E**) *p*MD2.

Copolymers, as opposed to the homopolymers, are chemically diverse which triggers different behavior for the adsorption of protein on the surface. The SEM images of *p*MMA, *p*MD1, *p*MD2, *p*MS1 and *p*MS2 of homopolymer and copolymers are shown in Fig. 6. Here SEM images, show the surface morphology of the *p*MMA and other copolymers of MMA with DMAEMA and SPMA after adsorption of BSA at 25 °C and pH 7.4. As *p*MMA has hydrophobic nature and showed greater BSA adsorption as compared to amphiphilic copolymers. In *p*MMA thick film of BSA is formed because of opposite charges on the surface of *p*MMA and BSA. On the other hand, amphiphilic copolymers *p*MD1, *p*MD2, *p*MS1 and *p*MS2 and BSA have negative charges and minimum adsorption was observed^46^.

Here both copolymers *p*MS1 and *p*MD1 have comparatively high concentration of hydrophilic monomers (3-sulfopropyl methacrylate and dimethyl aminoethyl methacrylate) than *p*MS2 and *p*MD2 which showed low adsorption of BSA. These copolymers have sulfate and amino functional that control their amphiphilic character and interaction of BSA with the polymers^26–30^.

### AFM analysis of copolymers

**Figure 7:**
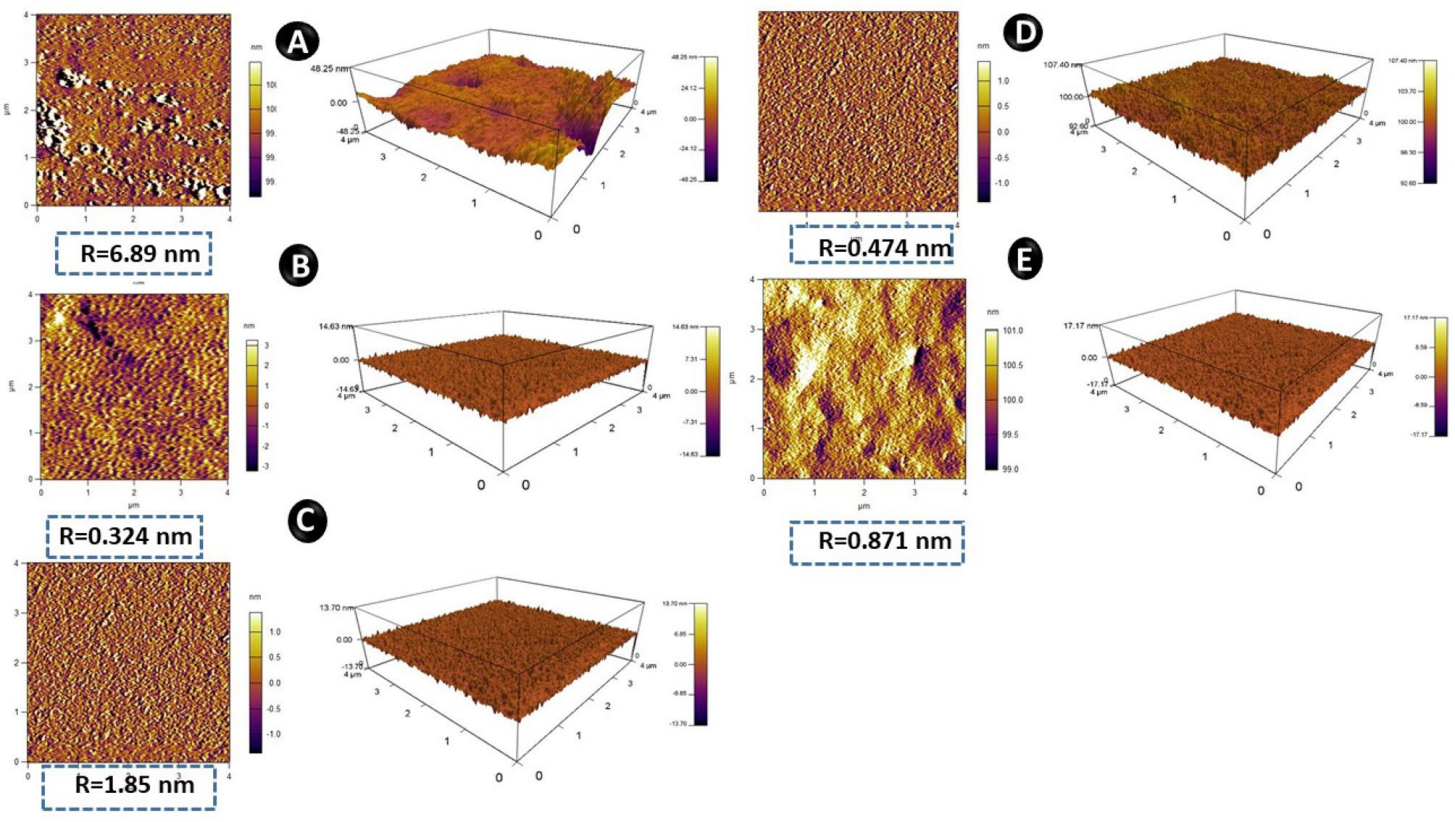
AFM analysis of (**A**) *p*MMA; (**B**) *p*MS1; (**C**) *p*MS2; (**D**) *p*MD1 and (**E**) *p*MD2.

AFM analysis of the polymers was performed at pH 7.4 in order to observe the film formation upon the surface of the polymers. BSA adsorption on the surface of polymers was performed and *p*MMA showed maximum roughness of 6.85 nm and least roughness of 0.374 nm and 0.474 was observed in *p*MS1 and *p*MD1, respectively (Table 2). *p*MMA has positive charge while *p*MD1, *p*MS1 and BSA have negative charges as observed through zeta potential. Because of similar charges amphiphilic copolymers showed minimum adsorption of BSA on the surface of polymers (Fig. 7).

Protein adsorption is directly related to the hydrophobic property of the material that reverses to the hydrophilic character of polymers. This observation supports the prior findings on homogeneous polymer surfaces that protein non-specific adsorption prefers more hydrophobic surfaces. This might be explained by the combined impact of the protein surface's amphiphilicity and the chemical heterogeneity of the copolymer interfaces. As a consequence, the total protein adsorption density increased with the density of chemical interfaces on the surface, and protein adsorbed on the *p*MMA surface was shown to be denser than that on the amphiphilic surfaces containing copolymers.

### Kinetic studies and adsorption modelling

**Figure 8:**
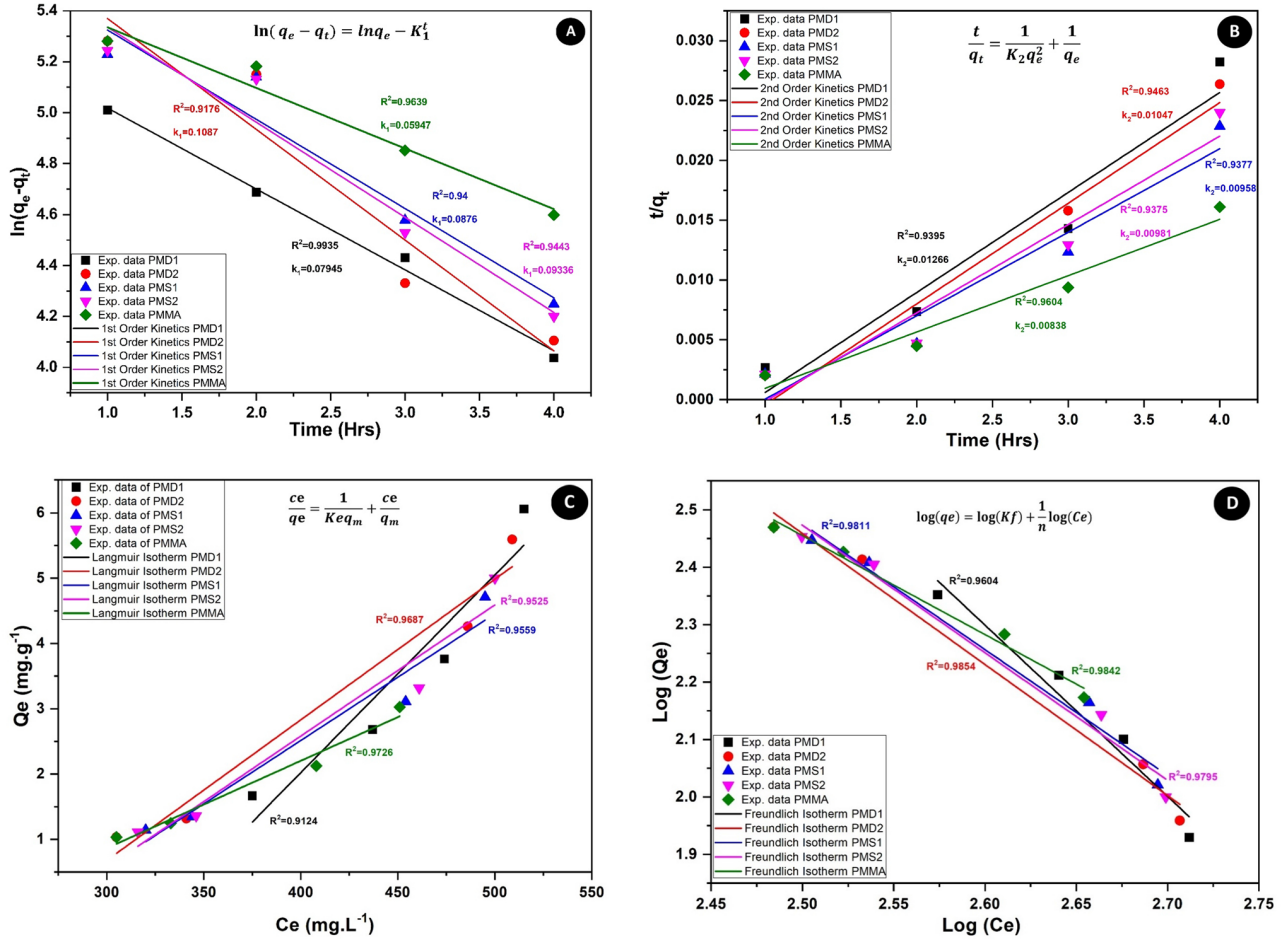
(**A**) Pseudo 1st order, (**B**) Pseudo 2nd order rate curves obtained from adsorption of BSA on polymers, (**C**) Langmuir isotherm and (**D**) Freundlich isotherm.

Adsorption kinetics for BSA was performed to check the adsorption phenomenon on the surface of polymers, Fig. 8A and B indicate the pseudo first and second order curves. The kinetic curves showed that initially the adsorption of BSA on the polymer was rapid that decreases with time. By comparing the R2 values of both pseudo first and second order for *p*MD1, *p*MD2, *p*MS1, *p*MS2 and *p*MMA, it is evident that all these polymers follow pseudo first order kinetics which means there is predominantly physisorption on the polymers. Moreover, the rate constant values k1 are very small in each polymer that indicates low adsorption possibility on the polymers. It is due to the antifouling behavior of these polymers as indicated in our previous studies^28,30,35^. These results are in accordance with the zeta potential values at pH 7.4 the BSA has a low negative charge as well as *p*MD1, *p*MD2, *p*MS1 and *p*MS2 also have a negative charge that causes repulsion to the incoming BSA molecules and results in low adsorption on the polymer surface.

*p*MMA has low positive charge and due to attraction BSA adsorbs on *p*MMA, hence it shows higher adsorption than the other amphiphilic copolymers^47–49^. Therefore, by copolymerizing MMA with DMAEMA and SPMA, the charge on the polymer changed to negative that results in the rejection of BSA, hence increasing the antifouling characteristic. The process of adsorption has also been expressed by Langmuir and Freundlich relations [Eqs. (1) and (2)] in Fig. 8C and D^50–52^. According to the data and the regression coefficient values, it is clear that the polymers follow the Freundlich isotherm which means that the adsorption of BSA takes place as the multilayer.

### Effect of time and BSA adsorption

**Figure 9:**
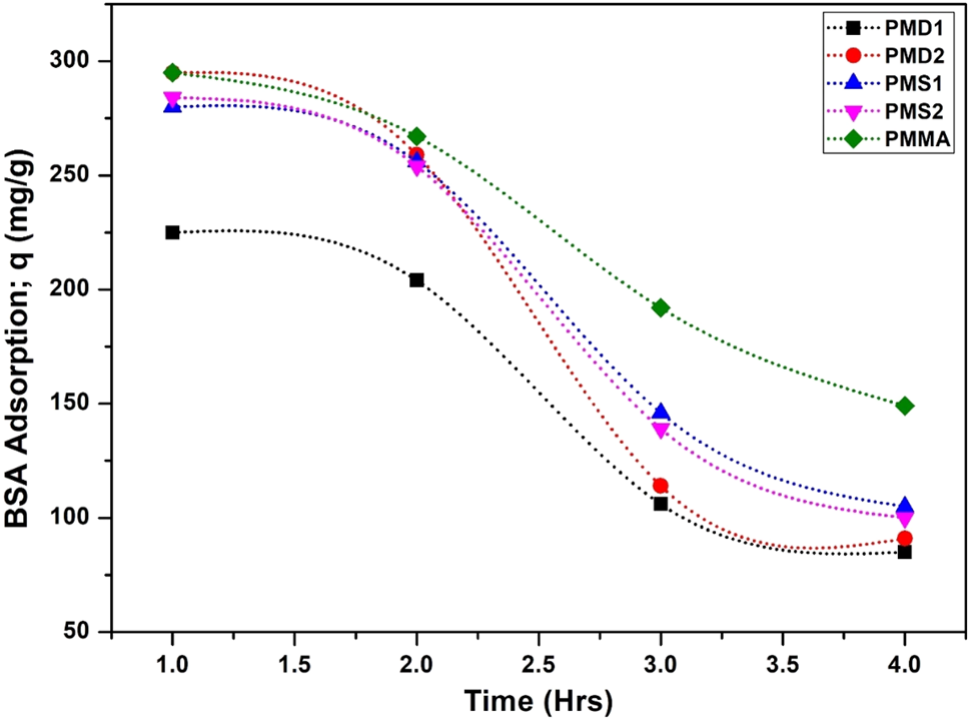
BSA adsorption on the surface of polymers with time.

Figure 9 represents the effect of time on the adsorption of BSA on polymers. Initially, there is a rapid adsorption of BSA onto the polymers. Adsorption greatly decreases as most of the adsorption sites were occupied initially, which lowers the adsorption of BSA and finally the steady state adsorption was achieved in 4 hrs^20^. Initially, 50–55% of BSA was rejected by the polymers, as time passed the adsorption sites were occupied and there was no further adsorption at the equilibrium so at this stage up to 80–85% of BSA was rejected. These results were significant as, 50–55% of BSA was rejected at the early stage that indicating the antifouling behavior of the polymers^53^. The maximum BSA adsorption, q in the case of pMD1 is 338.7, pMD2 is 331, pMS1 is 291, pMS2 is 301 and pMMA is 459 mg.g^−1^.

## Conclusion

In this study the homopolymer, *p*MMA and copolymers *p*(MMA-co-SPMA), *p*(MMA-co-DMAEMA) were synthesized with various composition. The copolymer *p*(MMA-co-SPMA) showed a better thermal stability than that of *p*(MMA-co-DMAEMA) and *p*MMA. Monomers concentration effected the thermal stability and glass transition temperature (*T*_g_) of copolymers, *p*MS1 and *p*MS2 were endowed with a higher heat distortion resistance by the introduction of SPMA in *p*MMA. Zeta potential measurements were performed at pH 7.4 to recognize the protein interactions on surface of polymers at room temperature. SEM and AFM analysis showed significant deposition of BSA protein on the surface of *p*MMA as compared to amphiphilic copolymers. In this study all the polymers followed the pseudo first order rate that showed physiosorption and Freundlich isotherm that indicates the multilayer adsorption. The adsorption of BSA decreases with time and the maximum adsorption was found in *p*MMA, while the copolymers showed lower adsorption than the *p*MMA that implies the higher antifouling characteristic of copolymer than *p*MMA.

## Data Availability

All data generated or analyzed during this study are included in this article.
